# Phenotypic Heterogeneity in Titinopathies with Peripheral Nerve Involvement in Pediatric Age: Two Case Reports

**DOI:** 10.3390/jcm15093552

**Published:** 2026-05-06

**Authors:** Carlo Alberto Cesaroni, Giulia Pisanò, Massimiliano Marton, Stefano Giuseppe Caraffi, Susanna Rizzi, Agnese Pantani, Diletta Ziveri, Marzia Pollazzon, Juha Koskenvuo, Daniele Frattini, Carlo Fusco

**Affiliations:** 1 Child Neurology and Psychiatry Unit, Mother and Child Department, Azienda USL-IRCCS di Reggio Emilia, 42123 Reggio Emilia, Italy; giulia.pisano@ausl.re.it (G.P.); massimarton@gmail.com (M.M.); susanna.rizzi@ausl.re.it (S.R.); agnese.pantani@ausl.re.it (A.P.); diletta.ziveri@gmail.com (D.Z.);; 2 Child Neurology and Psychiatry Unit, Pediatric Neurophysiology Laboratory, Mother and Child Department, Azienda USL-IRCCS di Reggio Emilia, 42123 Reggio Emilia, Italy; 3 Medical Genetics Unit, Mother and Child Department, Azienda USL-IRCCS di Reggio Emilia, 42123 Reggio Emilia, Italy; stefanogiuseppe.caraffi@ausl.re.it (S.G.C.); marzia.pollazzon@ausl.re.it (M.P.); 4 Blueprint Genetics, a Quest Diagnostics Company, 02150 Espoo, Finland; juha.koskenvuo@blueprintgenetics.com

**Keywords:** *TTN*, neurophysiology, electromyography, pain, neuromuscular disease, truncating variant, whole-exome sequencing, cardiomyopathy

## Abstract

**Background/Objectives:** Titin (TTN; OMIM 188840) is the largest known human sarcomeric protein, and pathogenic variants in the TTN gene cause a broad spectrum of inherited myopathies and cardiomyopathies. The extent to which TTN variants may also involve the peripheral nervous system remains poorly defined. We aimed to describe two pediatric patients carrying heterozygous truncating TTN variants with neurophysiological evidence of peripheral nerve involvement, and to review the existing literature on this underrecognized association. Pathogenic variants in the *TTN* gene are associated with a wide spectrum of inherited myopathies and cardiomyopathies. To date, peripheral neur opathy has not been recognized as a defining feature of *TTN*-related disorders, and neurophysiological investigations in affected individuals typically demonstrate normal or myopathic findings without evidence of a primary neuropathic process. Here, we report two pediatric patients with heterozygous truncating *TTN* variants and neurophysiological evidence of bilateral axonal involvement of the deep peroneal nerve. **Methods:** This case report was structured and reported according to the CARE guidelines. Genetic testing was performed using whole-exome sequencing (Blueprint Genetics Whole Exome Family Test). Nerve conduction studies and needle electromyography were performed using the Galileo NT system. Variant classification followed current ACMG guidelines. **Results:** The first patient, a 10-year-old girl, presented with a symptomatic distal motor phenotype characterized by bilateral pes cavus, anterior compartment muscle atrophy, areflexia, and steppage gait with onset in early childhood. The second patient, an 8-year-old boy, had subclinical bilateral axonal neuropathy identified during neurophysiological evaluation prompted by intermittent lower limb pain; his father, carrying the same variant, showed concordant neurophysiological abnormalities. In both cases, nerve conduction studies demonstrated reduced compound muscle action potential amplitudes with preserved conduction velocities and distal latencies, consistent with axonal neuropathy. Whole-exome sequencing excluded other established genetic causes of inherited neuropathy in both probands. **Conclusions:** Although a causal relationship cannot be established, these observations raise the possibility that peripheral nerve involvement may represent an underrecognized feature of the titinopathy spectrum. Prospective studies in larger cohorts of *TTN* variant carriers are needed to clarify the prevalence and pathophysiological basis of neuropathy in this context.

## 1. Introduction

Titin is the largest known protein in the human body, constituting the third myofilament of the sarcomere and spanning from the Z-disk to the M-band [1]. It provides passive elasticity to striated muscle and contributes to sarcomere assembly, force transmission, and mechanosensing. The elastic I-band region generates passive tension essential for maintaining sarcomere integrity and, in cardiac muscle, for regulating diastolic filling [2,3].

The *TTN* gene (OMIM 188840) encodes titin and undergoes extensive alternative splicing, giving rise to multiple tissue-specific isoforms. In cardiac muscle, at least three major isoform classes are expressed—fetal cardiac titin, adult N2BA, and adult N2B—which differ in their elastic properties and developmental regulation [4]. In skeletal muscle, the predominant isoforms vary by fiber type and developmental stage, contributing to the broad phenotypic spectrum of *TTN*-related disorders.

Pathogenic variants in *TTN* represent one of the most common genetic causes of both inherited cardiomyopathies and skeletal myopathies. Truncating *TTN* variants account for approximately 25% of familial dilated cardiomyopathy cases and are increasingly recognized in other cardiomyopathy subtypes [5,6,7]. In skeletal muscle, the recognized phenotypic spectrum includes tibial muscular dystrophy (Udd myopathy), early adult-onset recessive distal titinopathy, limb-girdle muscular dystrophy type R10 (formerly 2J), congenital centronuclear myopathy, early-onset myopathy with fatal cardiomyopathy, multi-minicore disease with cardiac involvement, Emery–Dreifuss-like phenotypes, hereditary myopathy with early respiratory failure (HMERF), and adult-onset recessive proximal muscular dystrophy [8]. In the pediatric population, titinopathies typically present with hypotonia, delayed motor milestones, and variable cardiac involvement, although the full phenotypic range in children remains incompletely characterized [9,10].

Despite this broad spectrum, peripheral neuropathy has not been recognized as a defining or consistent feature of *TTN*-related disorders. Neurophysiological investigations in titinopathy patients typically demonstrate myopathic rather than neuropathic findings, and the few reports suggesting a possible link between *TTN* variants and nerve involvement remain anecdotal and mechanistically unexplored.

Here, we report two pediatric patients carrying heterozygous truncating *TTN* variants who both demonstrated neurophysiological evidence of bilateral axonal involvement of the deep peroneal nerve. We discuss the potential clinical and pathophysiological significance of these findings in the context of the expanding phenotypic spectrum of titinopathies. The objective of this case report is to describe the clinical, neurophysiological, and genetic features of these two patients, and to discuss the potential significance of peripheral nerve involvement within the expanding phenotypic spectrum of TTN-related disorders.

## 2. Materials and Methods

This manuscript is a case report structured and reported in accordance with the CARE (CAse REport) guidelines. The study design is observational and descriptive, based on prospectively collected clinical, neurophysiological, and genetic data from two pediatric patients evaluated at our center.

### 2.1. Genetic Testing and Variant Classification

Genetic testing was performed at Blueprint Genetics (Keilaranta 16 A-B, 02150 Espoo, Finland) on genomic and mitochondrial DNA extracted from peripheral blood leukocytes of the probands and their parents using standard procedures. The Blueprint Genetics Whole Exome Family Test targets all protein-coding exons, exon–intron boundaries (±20 bp), and selected disease-associated deep intronic variants. Variant classification followed the American College of Medical Genetics and Genomics (ACMG) guidelines [11].

For Patient 1, the variant NM_001256850.2:c.50646del, p.(Pro16883Leufs3) was classified as Pathogenic based on the following ACMG criteria: PVS1 (null variant in a gene where loss-of-function is a known disease mechanism), PM2 (absent from gnomAD and RGC-MEV population databases), and PM6 (assumed de novo, confirmed by parental testing). For Patient 2, the variant NM_001256850.2:c.12268C>T, p.(Gln4090) was classified as Likely Pathogenic based on: PVS1, PM2, and PP1 (co-segregation with affected father) [11].

### 2.2. Neurophysiological Assessment

Nerve conduction studies (NCS) and needle electromyography (EMG) were performed using the Galileo NT system (EB Neuro S.p.A., Florence, Italy). NCS were conducted according to standard protocols with skin temperature maintained above 32 °C in the upper limbs and above 30 °C in the lower limbs to ensure reliable conduction velocity measurements. Reference values for compound muscle action potential (CMAP) amplitude of the deep peroneal nerve were defined as >3 mV when recording from the extensor digitorum brevis muscle; reduced amplitude was defined as a CMAP below this threshold with preserved distal latency and conduction velocity, consistent with axonal involvement [12]. Sensory nerve action potential (SNAP) amplitudes and conduction velocities were interpreted according to age-adjusted normative values.

Needle EMG was performed using a concentric needle electrode. The tibialis anterior muscle was examined bilaterally in both patients; additional muscles, including proximal limb muscles, were examined where clinically indicated. Spontaneous activity, motor unit action potential (MUAP) morphology (amplitude, duration, and phases), and recruitment patterns were assessed according to standard criteria.

### 2.3. Ethical Statement

Written informed consent was obtained from the parents of both patients prior to all diagnostic procedures and for the publication of this report. Given the purely observational and retrospective nature of this case report, formal ethics committee approval was not required under applicable institutional regulations (Azienda USL-IRCCS di Reggio Emilia, Italy); this exemption is consistent with national guidelines for case reports involving standard-of-care diagnostic procedures. Specific written consent for the use of whole-exome sequencing (WES) data, including genetic variants identified in the probands and family members, was obtained from the parents as part of the Blueprint Genetics clinical testing consent process. All data have been anonymized in accordance with the Declaration of Helsinki (revised 2013).

## 3. Results

### 3.1. Clinical Description

#### 3.1.1. Case Report 1

The first patient is a 10-year-old girl born at term after an uncomplicated pregnancy, with no family history of neuromuscular or neuropsychiatric disorders. Psychomotor development was within normal limits. From early childhood, gait was characterized by difficulty lifting the feet during the swing phase, consistent with bilateral steppage gait.

At the age of 9 years, progressive gait instability and increased frequency of falls prompted referral to our center. General physical examination was unremarkable. Neurological examination of the lower limbs revealed areflexia, Achilles tendon contracture, atrophy of the anterior compartment muscles, bilateral pes cavus, and reduced ankle dorsiflexion strength. Upper limb neurological examination was normal. Serum creatine kinase (CK) levels were within the normal range for age.

**Figure 1:**
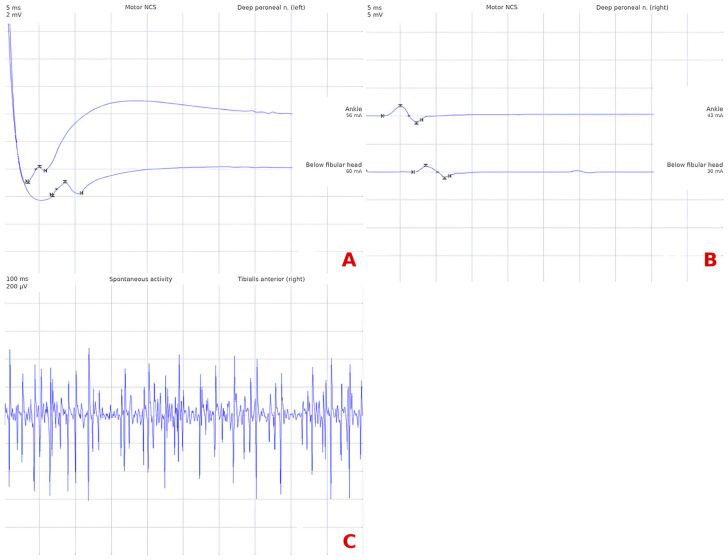
Nerve conduction studies of the deep peroneal nerve in Patient 1. (**A**) shows the left deep peroneal nerve and (**B**) shows the right deep peroneal nerve. Both sides demonstrate markedly reduced compound muscle action potential (CMAP) amplitudes at ankle stimulation, with preserved distal latencies and conduction velocities, consistent with bilateral axonal involvement. Numerical values are reported in Table 1. Nerve conduction parameters of all other examined nerves (left median motor and sensory, bilateral posterior tibial, and bilateral sural) were within normal limits. (**C**) shows needle electromyography of right tibialis anterior muscle. The trace shows spontaneous activity at rest, with continuous low-amplitude potentials (≤200 µV), irregular in distribution and density.

Nerve conduction studies demonstrated bilaterally reduced compound muscle action potential (CMAP) amplitudes of the deep peroneal nerve, with preserved distal latencies and conduction velocities, consistent with axonal involvement (Figure 1, Table 1). Motor and sensory nerve conduction parameters of all other examined nerves—including the median, posterior tibial, and sural nerves bilaterally—were within normal limits. Needle electromyography of the tibialis anterior muscle revealed myotonic-like discharges on the left side, with low-amplitude, short-duration motor unit action potentials and reduced recruitment pattern during maximal voluntary contraction, suggesting active myopathic involvement. On the right side, motor unit action potentials showed variable amplitudes with a similarly simplified recruitment pattern.

Multiplex ligation-dependent probe amplification (MLPA) testing for spinal muscular atrophy (*SMN1* deletion) was negative. Whole-exome sequencing, extended to both parents, identified a novel heterozygous truncating *TTN* variant in the proband: NM_001256850.2:c.50646del, p.(Pro16883Leufs*3), classified as pathogenic according to ACMG criteria. The variant was absent in both parents, consistent with a de novo origin. Residue Pro16883 maps to the distal I-band region, within or immediately proximal to the PEVK segment. The variant was absent from gnomAD (v4.1.1) and from the RGC Million Exome Variant Browser (v1.1.3; ~983,578 individuals), consistent with an ultra-rare allele frequency (PM2 criterion). Electrocardiogram and transthoracic echocardiography were normal, with no evidence of cardiomyopathy or conduction abnormalities.

**Figure 2:**
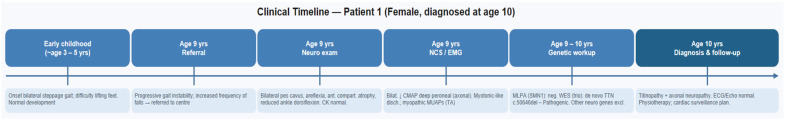
Clinical Timeline of Patient 1.

Clinical Timeline—Patient 2 is shown in Figure 2.

#### 3.1.2. Case Report 2

The second patient is an 8-year-old boy referred for evaluation of a behavioural disorder. In addition to the behavioural complaints, the patient reported a history of intermittent bilateral lower limb pain, without a clear precipitating factor. General physical examination was unremarkable. Neurological examination demonstrated normal muscle tone, bulk, and strength, with preserved and symmetrical deep tendon reflexes in all four limbs. No skeletal deformities or signs of anterior compartment muscle atrophy were observed.

As part of a diagnostic work-up to exclude genetic or metabolic causes of the behavioural phenotype, whole-exome sequencing was performed and revealed a novel heterozygous truncating *TTN* variant: NM_001256850.2:c.12268C>T, p.(Gln4090*), classified as likely pathogenic according to ACMG criteria. Segregation analysis demonstrated paternal inheritance of the variant. Residue Gln4090 maps to the proximal I-band region, in the vicinity of the N2B unique sequence. The variant was absent from gnomAD (v4.1.1) and from the RGC Million Exome Variant Browser (v1.1.3; ~983,578 individuals), consistent with an ultra-rare allele frequency (PM2 criterion).

**Figure 3:**
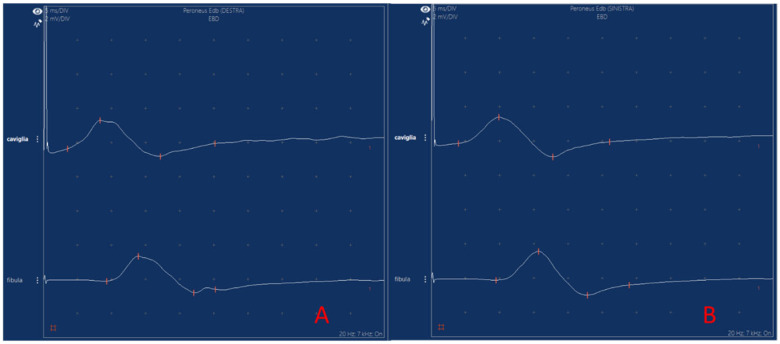
Nerve conduction studies of the deep peroneal nerve in Patient 2. (**A**) shows the right deep peroneal nerve; (**B**) shows the left deep peroneal nerve. Both sides demonstrate reduced compound muscle action potential (CMAP) amplitudes at ankle stimulation, with preserved distal latencies and conduction velocities, consistent with bilateral axonal involvement. Numerical values are reported in Table 1. Nerve conduction parameters of all other examined nerves (left median motor and sensory, left ulnar motor, bilateral posterior tibial, and bilateral sural) were within normal limits.

Given the reported lower limb pain, nerve conduction studies were subsequently performed. NCS revealed bilaterally reduced CMAP amplitudes of the deep peroneal nerve, with preserved distal latencies and conduction velocities, consistent with axonal involvement (Figure 3, Table 1). Motor and sensory nerve conduction parameters of all other examined nerves were within normal limits. Serum creatine kinase levels were within the normal range for age.

Neurophysiological evaluation of the father, who carried the same heterozygous *TTN* variant (NM_001256850.2:c.12268C>T, p.(Gln4090*)), demonstrated unilateral axonal involvement of the left deep peroneal nerve on NCS. Needle electromyography of the left tibialis anterior muscle showed motor unit action potentials of reduced amplitude and duration, consistent with myopathic changes. The father reported no significant neuromuscular complaints. Cardiac evaluation of the father, including 12-lead electrocardiogram and transthoracic echocardiography, was normal, with no evidence of cardiomyopathy or conduction abnormalities, consistent with a subclinical or oligosymptomatic neuromuscular phenotype without cardiac involvement at the time of evaluation.

Cardiac evaluation of the patient, including 12-lead electrocardiogram and transthoracic echocardiography, was normal, with no evidence of cardiomyopathy or conduction abnormalities.

**Figure 4:**
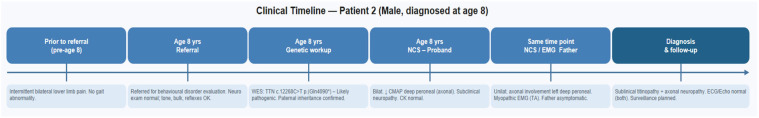
Clinical Timeline of Patient 2.

Clinical Timeline—Patient 2 is shown in Figure 4.

**Figure 5:**
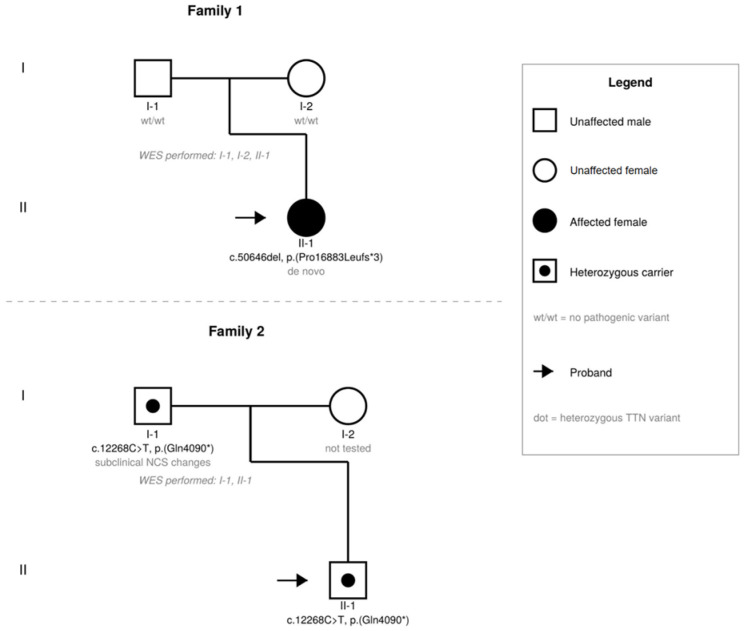
Pedigrees of Family 1 (I, II) and Family 2 (I, II). Filled circle: affected female; square/circle with central dot: heterozygous *TTN* variant carrier; arrow: proband; wt/wt: no pathogenic variant on whole-exome sequencing.

Pedigrees of both families are shown in Figure 5.

## 4. Discussion

Pathogenic variants in the *TTN* gene are well established as a major cause of both skeletal myopathies and inherited cardiomyopathies [5,8]. However, peripheral neuropathy has not been recognized as a defining or consistent feature of titinopathies.

A narrative review of the literature was performed by searching PubMed (last accessed: March 2026) using the following terms: TTN or titin combined with neuropath*, axonal, peripheral nerve, nerve conduction, and electrophysiol*. Additional sources were identified through manual screening of reference lists. Case reports, case series, and cohort studies reporting electrophysiological or clinical data on peripheral nerve function in TTN variant carriers were considered eligible.

In published cohorts, nerve conduction velocities are typically normal and EMG findings are either normal or myopathic, with no evidence of a primary neuropathic process [9,10].

Consistently, in a recent single-center cohort of 105 individuals with arthrogryposis multiplex congenita, *TTN* was the most frequently implicated gene in the myogenic subgroup, accounting for 23% of genetically resolved myogenic cases, while no *TTN*-related cases were classified in the neurogenic subgroup [13]. These observations reinforce the notion that peripheral nerve involvement is not an expected feature of titinopathy, making the neurophysiological findings in our patients particularly noteworthy.

In the present report, both patients showed neurophysiological evidence of axonal involvement of the deep peroneal nerve, albeit in markedly different clinical contexts. In the first patient, the findings were obtained in the setting of a clear distal motor phenotype, characterized by bilateral pes cavus, anterior compartment muscle atrophy, and areflexia—a presentation partially overlapping with tibial muscular dystrophy (Udd myopathy), an autosomal dominant distal myopathy originally described in the Finnish population and caused by heterozygous truncating variants in the terminal exons of *TTN* [14]. However, the early pediatric onset and the presence of symmetric axonal neuropathy are atypical features that distinguish this case from classical Udd myopathy, which typically manifests in the fourth to sixth decade of life. In the second patient, nerve conduction studies were prompted by intermittent lower limb pain and revealed subclinical bilateral axonal changes in an otherwise neurologically unremarkable child. Notably, the father—carrying the same heterozygous *TTN* variant—showed unilateral peroneal axonal involvement on NCS and myopathic EMG changes in the left tibialis anterior, a finding consistent with intrafamilial phenotypic variability and variable expressivity within the same family.

In both cases, the neuropathy was purely axonal in nature, with reduced CMAP amplitudes and preserved distal latencies and conduction velocities, consistent with a non-demyelinating, axonal process [12]. Comprehensive whole-exome sequencing excluded other established genetic causes of inherited peripheral neuropathy in both probands.

An important differential diagnosis to consider in cases of isolated deep peroneal nerve involvement is focal compressive neuropathy at the fibular head, which may result from habitual leg crossing, prolonged squatting, or sustained external pressure. Several features in the present cases argue against a purely compressive etiology: the strictly bilateral and symmetric distribution of the neurophysiological abnormalities, the absence of any history consistent with sustained positional compression, and the coexistence of myopathic changes on needle EMG. Nevertheless, a compressive contribution cannot be definitively excluded in the absence of prospective neuroimaging or serial neurophysiological assessments.

The clinical overlap between titinopathy and peripheral neuropathy is not merely theoretical: in a retrospective audit of 38 patients with distal myopathy, 18% had received an initial misdiagnosis of neuropathy before being reclassified, including at least one patient subsequently found to carry a pathogenic *TTN* variant [15]. These observations underscore the diagnostic challenge posed by distal titinopathies and highlight the importance of systematic neurophysiological assessment in *TTN* variant carriers, even when a primary neuropathic process is not clinically suspected.

The biological plausibility of a link between *TTN* variants and peripheral nerve involvement is supported by several converging lines of evidence, although all remain preliminary. First, *TTN* expression has been demonstrated not only in striated muscle but also in neurons of both the central and peripheral nervous systems [16].

Second, titin has been implicated in neurodegenerative processes—particularly in the context of amyotrophic lateral sclerosis—through mechanisms involving nucleolar stress and motor neuron vulnerability. Titin has been identified as a modifier of nucleolar stress pathways implicated in ALS neurodegeneration, suggesting a potential role in motor neuron biology beyond its structural function in muscle [17,18,19,20].

Third, experimental studies have demonstrated that titin fragments interact with filamentous actin and modulate actin polymerization [21]; given the central role of actin dynamics in axonal growth cone navigation and cytoskeletal integrity, disruption of titin function could theoretically impair axonal maintenance or development [21]. Whether any of these mechanisms is operative in the patients described here remains speculative.

The present observations are further supported by the identification of additional cases in the published literature. Luo et al. reported a patient with HMERF carrying a heterozygous *TTN* missense variant in exon 344 who demonstrated objective electrophysiological evidence of sensorimotor axonal polyneuropathy on NCS and EMG—the first such documentation in the HMERF phenotype, in a context where peripheral nerve involvement had previously been considered absent in over 100 reported cases [22]. More recently, Pérez-Arzola et al. described a patient with autosomal recessive LGMDR10 who showed left femoral axonotmesis and mixed axonal neuropathy of the upper limbs on electrodiagnostic evaluation, although a compressive etiology for the femoral neuropathy cannot be definitively excluded [23]. Taken together, these cases—spanning different *TTN*-related phenotypes, inheritance patterns, and age groups—suggest that peripheral nerve involvement in titinopathy may be more heterogeneous and underrecognized than previously appreciated. All identified cases with neurophysiological data are summarized in Table 2.

Finally, truncating *TTN* variants carry well-established implications for dilated cardiomyopathy risk [5], underscoring the importance of cardiac surveillance and long-term cardiology follow-up in all variant carriers, including currently asymptomatic individuals.

Of note, it has been demonstrated that the co-occurrence of truncating *TTN* variants and pathogenic *RBM20* variants is associated with a severe and early-onset form of dilated cardiomyopathy [24,25]. In both patients reported here, whole-exome sequencing did not identify pathogenic or likely pathogenic variants in *RBM20* or in other cardiomyopathy-associated genes. While cardiac evaluation was normal at the time of assessment, long-term cardiac surveillance remains indicated given the known penetrance trajectory of TTN truncating variants.

Limitations. Several limitations of this report must be acknowledged. First, the small sample size—limited to two cases—precludes any inference about prevalence, causal attribution, or generalizability of these findings to larger cohorts of *TTN* variant carriers. Second, the observational and cross-sectional nature of the evaluation means that longitudinal neurophysiological data are unavailable; it is therefore not possible to determine whether the axonal changes observed are progressive, stable, or incidental. Third, while WES excluded alternative genetic causes of inherited neuropathy, a compressive etiology for deep peroneal nerve involvement—in particular from habitual leg crossing or sustained positional pressure—cannot be definitively ruled out in the absence of prospective neuroimaging or serial electrophysiological assessments. These constraints underscore the need for prospective, multicenter studies specifically designed to assess peripheral nerve involvement in titinopathy.

## 5. Conclusions

We report two pediatric patients with heterozygous truncating *TTN* variants and objective neurophysiological evidence of bilateral axonal involvement of the deep peroneal nerve. The clinical presentations ranged from a symptomatic distal motor phenotype to a subclinical neurophysiological finding, highlighting the phenotypic heterogeneity that may be associated with *TTN*-related disorders. Although a direct causal relationship cannot be established on the basis of these observations alone, the findings raise the possibility that peripheral nerve involvement—whether primary or secondary—may represent an underrecognized feature of the titinopathy spectrum. Prospective neurophysiological studies in larger cohorts of *TTN* variant carriers will be essential to determine the prevalence, clinical significance, and pathophysiological basis of neuropathy in this context.

## Figures and Tables

**Table 1:** Nerve conduction study results of the deep peroneal nerve (bilateral) in Patient 1 and Patient 2. Asterisks (*) indicate CMAP amplitudes below the normal reference value (>3 mV). EDB = extensor digitorum brevis; CV = conduction velocity; CMAP = compound muscle action potential.

Nerve	Site/Segment	Latency (ms)	Amplitude (mV)	Distance (mm)	CV (m/s)
Patient 1—reference values: CMAP > 3 mV; CV > 40 m/s; distal latency < 4.5 ms					
Left deep peroneal	Ankle—EDB	3.5	1.0 *	—	—
	Below fibular head—Ankle	6.9	0.877 *	200	58.9
Right deep peroneal	Ankle—EDB	2.8	2.7 *	—	—
	Below fibular head—Ankle	7.0	2.0 *	222	52.2
Patient 2—reference values: CMAP > 3 mV; CV > 40 m/s; distal latency < 4.5 ms					
Left deep peroneal	Ankle—EDB	3.97	2.32 *	—	—
	Fibula—Ankle	9.46	2.57 *	300	53.93
Right deep peroneal	Ankle—EDB	3.56	2.12 *	—	—
	Fibula—Ankle	9.29	2.14 *	300	52.36

Normal reference values (age-adjusted): deep peroneal nerve CMAP amplitude > 3 mV; conduction velocity > 40 m/s; distal latency < 4.5 ms.

**Table 2:** Published cases of *TTN* variant carriers with documented peripheral nerve involvement on neurophysiological evaluation.

Patient	Age/Sex	TTN Variant	Titin Region	Inheritance	Clinical Phenotype	NCS Findings	EMG Findings	Other Genes Excluded	Reference
Patient 1	10 y/F	NM_001256850.2: c.50646del, p.(Pro16883Leufs*3)	Distal I-band (PEVK region)	AD (de novo)	Bilateral pes cavus, anterior compartment atrophy, areflexia, steppage gait	Reduced CMAP bilateral deep peroneal nerve; preserved DL and CV	Myotonic-like discharges, low-amplitude short-duration MUAPs, reduced recruitment left TA; variable MUAPs simplified recruitment right TA	WES excluded other inherited neuropathy genes	Present series
Patient 2	8 y/M	NM_001256850.2: c.12268C>T, p.(Gln4090*)	Proximal I-band (N2B region)	AD (paternal)	Subclinical; intermittent lower limb pain. Father (same variant): unilateral peroneal axonal involvement and myopathic EMG changes	Proband: reduced CMAP bilateral deep peroneal nerve; preserved DL and CV. Father: reduced CMAP left deep peroneal nerve; preserved DL and CV	Proband: not performed. Father: low-amplitude short-duration MUAPs left TA (myopathic pattern)	WES excluded other inherited neuropathy genes	Present series
Case 3	42 y/M	NM_001267550.1: c.95134T>C, p.(Cys31712Arg), exon 344	A-band (FnIII-119/A150 domain)	AD	HMERF: distal lower limb weakness, early respiratory failure	Reduced CMAP and SNAP amplitudes; preserved CV (axonal sensorimotor pattern)	Fibrillation potentials; large MUAPs	Not specified	Luo et al. [22]
Case 4	39 y/M	NM_001267550.1: c.107578C>T, p.(Gln37860*) + c.104269C>T, p.(Gln34767*)	Distal A-band/M-line	AR (compound het.)	LGMDR10: proximal asymmetric weakness, left leg atrophy, myalgia	Left femoral neuropathy (axonotmesis pattern); mixed axonal neuropathy upper limbs *	Myopathic pattern upper limbs	Not specified	Pérez-Arzola et al. [23]

* A compressive etiology for the left femoral neuropathy cannot be excluded in this case. Abbreviations: AD, autosomal dominant; AR, autosomal recessive; CMAP, compound muscle action potential; CV, conduction velocity; DL, distal latency; EMG, electromyography; F, female; het., heterozygous; HMERF, hereditary myopathy with early respiratory failure; LGMDR10, autosomal recessive limb-girdle muscular dystrophy type 10; M, male; MUAP, motor unit action potential; NCS, nerve conduction studies; SNAP, sensory nerve action potential; TA, tibialis anterior; WES, whole-exome sequencing; y, years. Variant nomenclature follows the reference transcript reported in the original publication.

## Data Availability

The data presented in this report are available from the corresponding author upon reasonable request, subject to applicable privacy regulations.
